# Expression of IL-1β in rhesus EAE and MS lesions is mainly induced in the CNS itself

**DOI:** 10.1186/s12974-016-0605-8

**Published:** 2016-06-06

**Authors:** Saskia Maria Burm, Laura Anna Norma Peferoen, Ella Alwine Zuiderwijk-Sick, Krista Geraldine Haanstra, Bert Adriaan ‘t Hart, Paul van der Valk, Sandra Amor, Jan Bauer, Jeffrey John Bajramovic

**Affiliations:** Alternatives Unit, Biomedical Primate Research Centre, Lange Kleiweg 161, 2288 GJ Rijswijk, The Netherlands; Department of Pathology, VU Medical Center, PO Box 7057, 1007 MB Amsterdam, The Netherlands; Department of Immunobiology, Biomedical Primate Research Centre, Lange Kleiweg 161, 2288 GJ Rijswijk, The Netherlands; Department of Neuroimmunology, Medical University of Vienna, Spitalgasse 4, A-1090 Vienna, Austria

**Keywords:** Multiple sclerosis, Experimental autoimmune encephalomyelitis, Inflammasome, IL-1β, Microglia, Preactive lesion

## Abstract

**Background:**

Interleukin (IL)-1β is a pro-inflammatory cytokine that plays a role in the pathogenesis of multiple sclerosis (MS) and experimental autoimmune encephalomyelitis (EAE), the animal model for MS. Yet, detailed studies on IL-1β expression in different stages of MS lesion development and a comparison of IL-1β expression in MS and EAE are lacking.

**Methods:**

Here, we performed an extensive characterization of IL-1β expression in brain tissue of MS patients, which included different MS lesion types, and in brain tissue of rhesus macaques with EAE.

**Results:**

In rhesus EAE brain tissue, we observed prominent IL-1β staining in MHC class II^+^ cells within perivascular infiltrates and at the edges of large demyelinating lesions. Surprisingly, staining was localized to resident microglia or differentiated macrophages rather than to infiltrating monocytes, suggesting that IL-1β expression is induced within the central nervous system (CNS). By contrast, IL-1β staining in MS brain tissue was much less pronounced. Staining was found in the parenchyma of active and chronic active MS lesions and in nodules of MHC class II^+^ microglia in otherwise normal appearing white matter. IL-1β expression was detected in a minority of the nodules only, which could not be distinguished by the expression of pro- and anti-inflammatory markers. These nodules were exclusively found in MS, and it remains to be determined whether IL-1β^+^ nodules are destined to progress into active lesions or whether they merely reflect a transient response to cellular stress.

**Conclusions:**

Although the exact localization and relative intensity of IL-1β expression in EAE and MS is different, the staining pattern in both neuroinflammatory disorders is most consistent with the idea that the expression of IL-1β during lesion development is induced in the tissue rather than in the periphery.

**Electronic supplementary material:**

The online version of this article (doi:10.1186/s12974-016-0605-8) contains supplementary material, which is available to authorized users.

## Background

IL-1β is a cytokine with potent pro-inflammatory characteristics. High levels of systemic IL-1β lead to a rise in body temperature by affecting the activity of the hypothalamus, to vasodilation, and to increased expression of adhesion factors on endothelial cells enabling transmigration of leukocytes [1, 2]. Furthermore, IL-1β orchestrates the innate immune response [3] and can induce skewing of T cells towards Th17 cells [4–7], thereby linking innate immune responses to activation of the adaptive immune system. The synthesis of IL-1β precursor protein is induced by IL-1α or by activation of receptors of the innate immune system such as Toll-like receptors (TLR) and NOD-like receptors (NLR) [8, 9]. Secretion of bioactive IL-1β requires additional cleavage of the precursor protein by a cysteine protease, which in turn requires activation [10]. Caspase 1 is the best-described cysteine protease that is activated by a protein complex called the inflammasome [10, 11].

Inflammasomes play a role in several neurodegenerative and neuroinflammatory diseases as well as in animal models for such diseases [12–18]. NLR-mediated activation is critically involved in inflammasome formation and is evoked by disturbances in cellular homeostasis, as caused by, e.g., pathogens, large protein aggregates, and neighboring cell death. Subsequently, NLR associate with inflammatory caspases, mostly via the adaptor protein ASC, leading to processing and secretion of pro-inflammatory cytokines such as IL-1β and IL-18 [19, 20].

The involvement of IL-1β and the inflammasome in experimental autoimmune encephalomyelitis (EAE), a commonly used animal model for MS, has been confirmed in different studies [21]. Inhibition of IL-1-induced signaling ameliorates the development of EAE in both rats and mice [22–25], and mice that are deficient in NLRP3, ASC, or caspase 1 expression are characterized by delayed onset of disease and less severe clinical symptoms [26–28]. Furthermore, expression levels of IL-1β [29–31], specific NLRs (e.g., NLRP1 and NLRP3) and caspase 1 are increased in the brain and spinal cord during disease [26, 32]. In addition, treatment with a caspase 1 inhibitor attenuates clinical signs of mouse EAE [33]. Treatment with interferon (IFN)β, a registered therapeutic biological for MS [34], decreases brain pathology by reducing serum IL-1β and caspase 1 activation levels [35].

In human macrophages, IFNβ inhibits inflammasome-mediated activation by inhibition of pro-IL1β transcription, by decreasing the availability of NLRP3-activating ligands, and by directly inhibiting NLRP3 and caspase-1 activation via post-translational modifications [35–37]. In line with this, monocytes derived from IFNβ-treated MS patients are characterized by decreased IL-1β production in response to inflammasome-activating stimuli [36]. More evidence for the involvement of IL-1β in MS pathogenesis comes from studies demonstrating that elevated IL-1β levels in cerebrospinal fluid (CSF) and blood of MS patients correlate with disease susceptibility, severity, and progression [38–43]. In addition, therapeutic approaches used for treatment of MS, i.e., IFNβ, Copaxone, or steroid treatment lead to increased levels of IL-1 receptor antagonist (IL-1RA), the natural inhibitor of the IL-1 receptor, in the blood [39, 44, 45].

Although these data suggest a role for IL-1β in both EAE and MS and provide a rationale for clinical trials that target the IL-1 axis [8], there are discordant results relating to the expression of IL-1β during the course of both diseases. While there is consensus on the abundant expression of IL-1β in the brain during EAE induced in rats and mice [29–31, 46], reports on IL-1β expression in MS lesions [47–49] are by no means unequivocal [50, 51]. Furthermore, it is unclear during which stages of pathogenesis IL-1β is produced and by which cells. We therefore characterized IL-1β expression in the brain tissue of MS patients, which included different types of MS lesions, side-by-side with brain tissue derived from rhesus macaques in which EAE was induced. We performed in depth analyses of the cellular sources of IL-1β expression and phenotyped these cells based on the expression of pro- and anti-inflammatory markers. Our results reveal distinct characteristics of either EAE or MS that might well reflect differences in pathogenesis. However, in both neuroinflammatory disorders, the expression of IL-1β during disease progression is mainly induced in the brain itself.

## Methods

### Brain tissue

We selected paraffin-embedded tissue blocks from three rhesus macaques without neurological disease, from eight rhesus macaques with EAE and from four immunized rhesus macaques that did not develop clinical disease (Table 1) from earlier studies [52–54] that were performed at the Biomedical Primate Research Centre (BPRC; Rijswijk, the Netherlands), and of which the tissue blocks were archived at the Department of Neuroimmunology from the Center of Brain Research (Vienna, Austria). As such no animals were sacrificed for the exclusive purpose of this study, thereby complying with the priority 3Rs program of the BPRC. EAE was induced by immunization with recombinant human (rh)MOG protein either in incomplete or complete Freund’s adjuvant (resp. IFA or CFA) [52–54]. All procedures were performed in compliance with guidelines of the Institutional Animal Care and Use Committee (IACUC) in accordance with Dutch law.

**Table 1:** Characteristics of the rhesus macaques

Animal ID	Gender	Age (year)	Weight (kg)	Immunizations (d)	EAE score ≥2 (d)	Euthanasia (d)	EAE score at euthanasia	Ref
Rhesus EAE								
R05045	M	8	10.7	rhMOG/CFA (0)	12	12	5	[54]
R06052	M	7	7.7	rhMOG/CFA (0)	13	13	5	[54]
R07035	M	6	10.0	rhMOG/CFA (0)	16	16	5	[54]
R06088	M	7	11.4	rhMOG/CFA (0)	17	18	5	[54]
R08043	M	5	8.1	rhMOG/CFA (0)	21	21	5	[54]
R06030	M	7	12.2	rhMOG/CFA (0)	26	27	5	[54]
Ri0106111	M	9	12.5	rhMOG/IFA (0, 28)	40	41	5	[53]
Ri970621	M	9	8.3	rhMOG/IFA (0, 28)	46	48	2.5	[53]
Controls								
R8765	F	11	9.5	–	–	–	–	
R02095	M	5	11.3	–	–	–	–	
R9222	F	17	9.3	–	–	–	–	
R97058	M	12	11.5	rhMOG/CFA (0)	–	22	0	[52]
R01097	M	9	13.9	rhMOG/CFA (0)	–	18	0	[52]
Ri9604157	M	14	8.2	rhMOG/IFA (0, 28, 56, 84)	–	111	0	[53]
Ri9805013	M	12	9.3	rhMOG/IFA (0, 28)	–	28	0	[53]

Human brain tissue samples were obtained from the Netherlands Brain Bank (NBB; coordinator Dr. Huitinga, Amsterdam, the Netherlands). NBB received permission to perform autopsies for the use of tissue and to access medical records for research purposes from the Medical Ethical Committee of the VU Medical Centre (Amsterdam, the Netherlands). All patients and controls, or their next of kin, had given informed consent for autopsy and the use of brain tissue for research purposes. Relevant clinical information was retrieved from the medical records and is summarized in Table 2. We selected a total of 45 tissue blocks (paraffin-embedded 22 blocks; frozen 23 blocks) from 28 MS patients (female-to-male ratio 4:3; average age 60.9 years; average post-mortem delay 8 h) and five tissue blocks from five donors without neurological disease (female-to-male ratio 2:3; average age 70.8 years; average post-mortem delay 6 h). This panel represented different types of MS, including relapsing remitting (RR), secondary progressive (SP), and primary progressive (PP) MS.

**Table 2:** Characteristics of the MS patients and controls

	Age (year)	Gender	PM delay (h)	MS type	Cause of death
MS cases					
1	44	F	10	PP	Decompensation
2	47	F	4	Unknown	Metastasis in the lung
3	57	F	8	RR	Sepsis
4	77	M	8	RR	Possible urosepsis
5	77	F	10	SP	Euthanasia
6	86	M	10	RR	Heart failure and pneumonia
7	43	M	8	Unknown	Pneumonia
8	66	F	6	Unknown	Unknown
9	48	F	11	Unknown	Hepatic encephalitis
10	48	F	5	PP	Euthanasia
11	54	M	8	PP	Euthanasia
12	56	M	10	PP	Cachexia and exhaustion by end stage MS
13	63	M	7	PP	Cardiac arrest
14	69	F	7	Unknown	Respiratory failure and heart failure
15	66	M	7	Unknown	Unknown
16	44	M	10	PP	Increasing pain control and halting food administration; possible infection
17	51	M	11	SP	Unknown
18	66	F	10	PP	Euthanasia
19	50	F	7	SP	Euthanasia
20	48	F	6	RR	Cardiac failure
21	49	M	8	SP	Pneumonia
22	60	F	10	SP	Euthanasia
23	61	M	9	SP	Euthanasia
24	76	F	9	Unknown	Unknown
25	84	F	<0.5	PP	Euthanasia
26	81	M	9	Unknown	General deterioration
27	66	F	6	SP	Metastasis in the liver
28	67	F	9	SP	Palliative sedation
Controls					
1	79	M	4	–	Dehydration by advanced multi-infarct dementia
2	56	M	9	–	Myocardial infarction
3	62	M	7	–	Unknown
4	84	F	6	–	Pneumonia
5	73	F	4	–	Renal insufficiency

### Immunohistochemistry

An overview of the used antibodies and their dilutions is given in Table 3. Isotype controls and omission of the primary antibodies were used to confirm specificity of the primary antibodies.

**Table 3:** Overview of used antibodies

	Source	Species	Dilution
Primary antibodies			
IL-1β (clone C-20)	Santa Cruz Biotechnology	Goat	Frozen: 1:100
			Parafin: 1:250
PLP (clone plpc1)	AbD Serotec	Mouse	Frozen: 1:500
			Parafin: 1:300
HLA-DR (clone LN3)	eBioscience	Mouse	Frozen: 1:750
			Parafin: 1:500
Iba-1	Wako	Rabbit	1:250
MRP14 (clone S36.48)	BMA Biomedicals	Mouse	1:100
CCL22	Abcam	Rabbit	1:100
CD200R (clone OX108)	AbD Serotec	Mouse	1:50
CD40 (clone LOB7/6)	AbD Serotec	Mouse	1:50
CD74 (clone By2)	Santa Cruz Biotechnology	Mouse	1:1600
MR (clone 19.2)	BD Pharmingen	Mouse	1:150
Isotype control	Southern Biotech	Goat	1:2500
Secondary antibodies			
EnVision HRP-labeled anti-mouse/rabbit polymer	DAKO	–	undiluted
Biotinylated anti-sheep/goat	Amersham	Donkey	1:200
Avidin-HRP	Sigma Aldrich	–	1:100
Anti-goat HRP	Jackson ImmunoResearch	Donkey	1:100
Anti-mouse IgG alkaline phosphatase	DAKO	Goat	1:250
Anti-rabbit IgG alkaline phosphatase	Southern Biotech	Goat	1:250
Avidin-CY2	Jackson ImmunoResearch	–	1:150
Anti-rabbit TRITC	Jackson ImmunoResearch	Donkey	1:100
Anti-mouse TRITC	Jackson ImmunoResearch	Donkey	1:50

Five micrometer-thick paraffin sections were collected on Superfrost Plus glass slides (VWR international, Leuven, Belgium) and dried at 37 °C. Tissue sections were characterized for the presence of demyelination by staining for proteolipid protein (PLP) and for inflammation by staining for MHC class II. PLP and MHC class II were stained according to a previously described protocol [55] using mouse anti-human PLP or mouse anti-human HLA-DR antibodies (these are also cross reactive to the rhesus macaque equivalent Mamu-DR), EnVision horse radish peroxidase (HRP), and 3,3′-diaminobenzidine (DAB; both DAKO, Heverlee, Belgium). Consecutive sections were stained for IL-1β as described previously [56] using goat anti-human IL-1β antibodies, biotinylated anti-sheep/-goat antibodies, avidin-HRP, and DAB.

Five micrometer-thick cryosections were collected on Superfrost Plus glass slides and air-dried. For IL-1β stainings, sections were formalin-fixed for 10 min, endogenous peroxidase was quenched in 0.3 % H_2_O_2_ in phosphate-buffered saline (PBS) and sections were incubated with 10 % fetal calf serum (FCS) in wash buffer (DAKO) for 20 min at RT. Thereafter, sections were incubated with goat anti-human IL-1β antibodies overnight at 4 °C. After rinsing, the primary antibody was reapplied for 1 h at RT, followed by incubation with donkey anti-goat HRP and they were developed with DAB.

Immunohistochemical double staining for IL-1β with MHC class II, CD74, CD40, CD200R, CCL22, or MR were performed on cryosections. Slides were stained for IL-1β as described above and developed with DAB. Thereafter, slides were rinsed thoroughly and incubated with anti-human HLA-DR, CD74, CD40, CD200R, CCL22, or MR antibodies overnight at 4 °C. Then slides were incubated with either goat anti-mouse IgG alkaline phosphatase or goat anti-rabbit IgG alkaline phosphatase and further developed with Liquid Permanent Red solution (Dako) for 10 min at RT.

Sections were imaged using the Olympus BX50 microscope and Canvas X Pro (Canvas X software Inc, 2015, version 16, build 2115) was used for graphical representations.

### Immunofluorescence

Immunofluorescent double staining for IL-1β with Iba-1 or MRP14 were performed on paraffin-embedded tissue sections. Antigen retrieval was performed by heating the slides in Tris-EDTA (pH 8.5). Thereafter, sections were incubated with anti-human IL-1β overnight at 4 °C. After rinsing the slides, the primary antibody was reapplied for 1 h at RT, followed by incubation with biotinylated anti-sheep/-goat antibodies and avidin-CY2 in the dark. Next, slides were incubated with anti-human Iba-1 or MRP14 antibodies for 1 h at RT. Then slides were incubated with either donkey anti-rabbit TRITC or donkey anti-mouse TRITC and embedded in Vectashield mounting medium containing DAPI (Brunswig chemie). Sections were imaged using the Nikon Microphot-FXA microscope and Canvas X Pro was used for graphical representations.

## Results

### Rhesus EAE

We studied brain tissue from three rhesus macaques without neurological disease and from 12 rhesus macaques that were immunized with rhMOG in either IFA or CFA, of which eight animals developed clinical EAE (Table 1). Brain tissue from control animals and from animals that did not develop clinical EAE did not contain detectable demyelination, inflammatory activity, or IL-1β. Tissue from animals that developed clinical EAE was characterized by perivascular infiltrates. We observed considerable inter-donor variability concerning the number and extent of the observed EAE lesions, probably attributable to the outbred nature of the model [54].

**Fig. 1:**
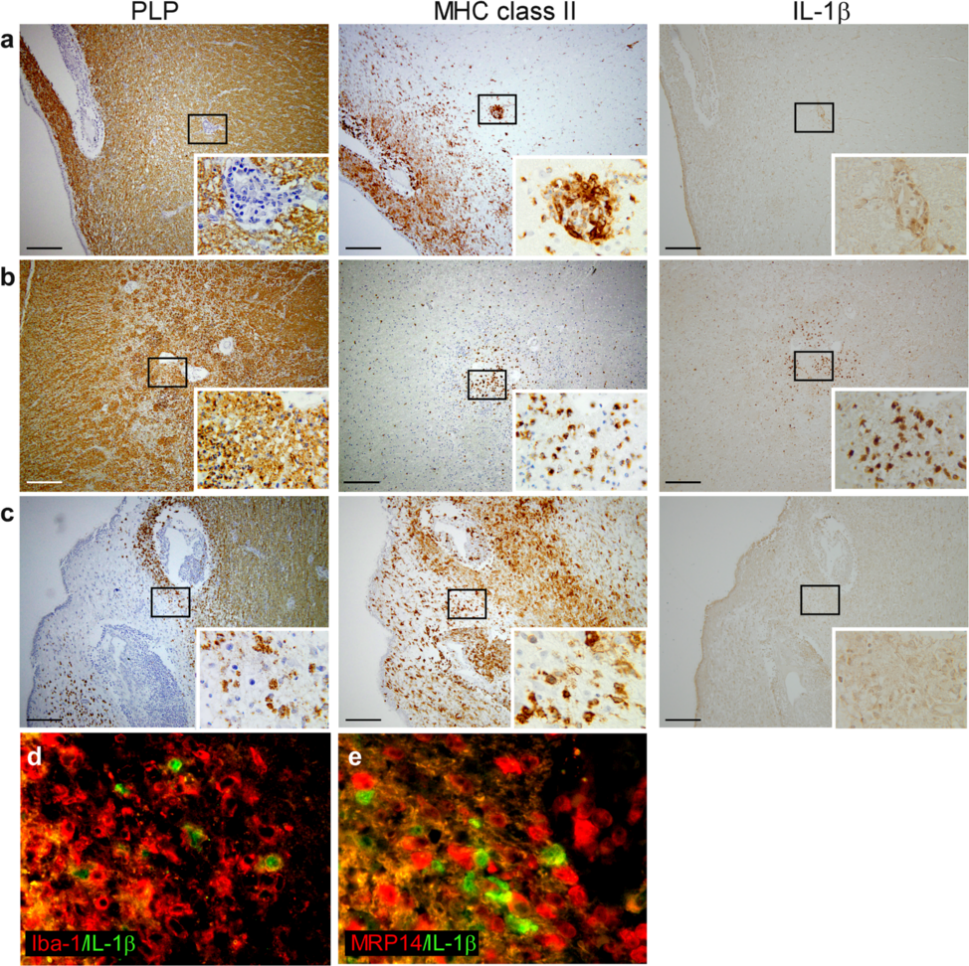
IL-1β expression in brain tissue of rhesus macaques with EAE induced by rhMOG in IFA. Brain lesions were characterized based on the extent of myelin (PLP in *brown, left panels*) damage and activation of innate immune cells (MHC class II in *brown*, *middle panels*). In small perivascular lesions without signs of demyelination (**a**), IL-1β staining (*in brown, right panels*) was mainly localized in MHC class II^+^ cells at the edge of the lesion. In mid-sized MHC class II^+^ lesions with clear signs of demyelination (**b**), IL-1β staining was more pronounced. In large fulminating lesions with extensive demyelination and infiltration of MHC class II^+^ cells (**c**), IL-1β staining was less pronounced compared to the mid-sized lesions and observed at the edge of the demyelinated area. Double labeling of perivascular lesions for IL-1β (*in green*) and Iba-1 or MRP14 (*in red*) demonstrated that all IL-1β^+^ cells were Iba-1^+^ (**d**), whereas all IL-1β^+^ cells were MRP14^−^ or MRP14^low^ (**e**). Original magnifications ×10, *scale bar* represents 200 μm, insets ×100. Nuclei were counterstained with hematoxylin (*blue*)

In animals immunized with rhMOG in IFA, we studied 30 perivascular lesions and three large areas with infiltrating cells and demyelination (Table 4). IL-1β staining was observed in 50 % of the perivascular infiltrates closely surrounding blood vessels (Fig. 1a, b) and in all large areas with extensive MHC class II expression and demyelination (Fig. 1c). Double immunofluorescent staining identified all IL-1β^+^ cells as Iba-1^+^ (Fig. 1d) and as MRP14^−^ or MRP14^low^ (Fig. 1e). As MRP14 is a marker that is strongly expressed on monocytes and neutrophils [57–59], this staining pattern is most consistent with microglia or differentiated macrophages as main sources of IL-1β.

**Table 4:** IL-1β expression in rhesus macaques with EAE

Animal code	Perivascular lesions		Large demyelinated areas with MHC class II^+^ cells	
	Total	IL-1β^+^	Total	IL-1β^+^
R05045	0	0	0	0
R06052	169	83	7	6
R07035	4	4	0	0
R06088	154	26	0	0
R08043	22	10	3	1
R06030	83	31	0	0
Total	432	154	10	7
Ri0106111	13	6	2	2
Ri970621	17	9	1	1
Total	30	15	3	3

**Fig. 2:**
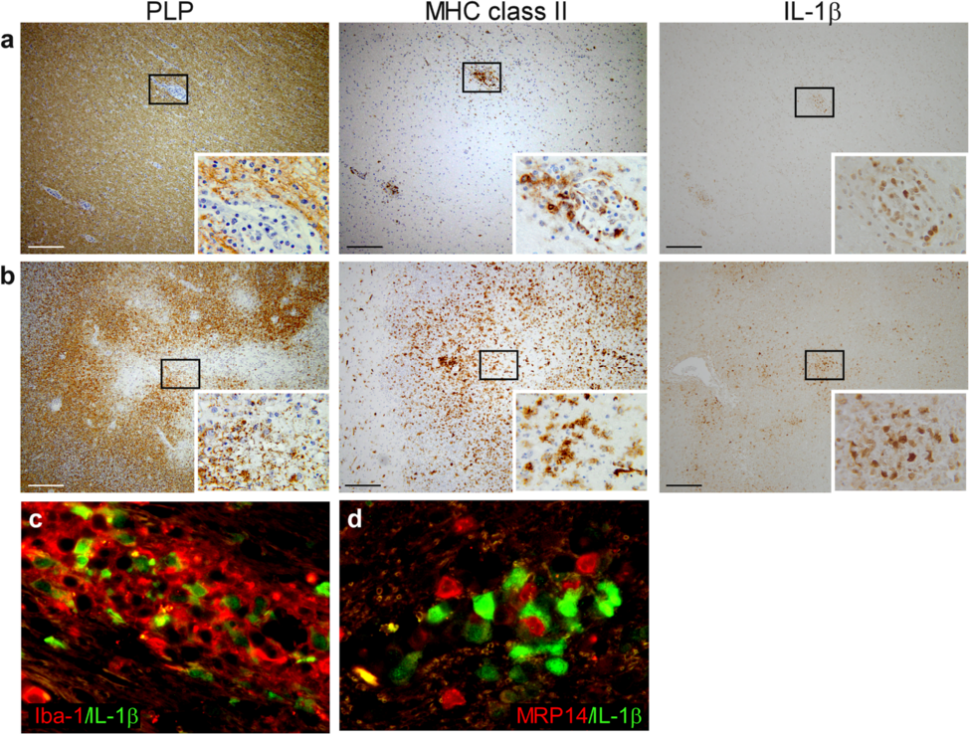
IL-1β expression in brain tissue of rhesus macaques with EAE induced by rhMOG in CFA. Brain lesions were characterized based on the extent of myelin (PLP *in brown, left panels*) damage and activation of innate immune cells (MHC class II *in brown, middle panels*). In small perivascular lesions without signs of demyelination (**a**), IL-1β staining (*in brown, right panels*) was mainly localized in MHC class II^+^ cells at the edge of the lesion. In large fulminating lesions with extensive demyelination and infiltration of MHC class II^+^ cells (**b**), IL-1β staining was more pronounced and mainly observed at the edges of the demyelinated area. Double labeling of perivascular lesions for IL-1β (*in green*) and Iba-1 or MRP14 (*in red*) demonstrated that all IL-1β^+^ cells were Iba-1^+^ (**c**), whereas all IL-1β^+^ cells were MRP14^−^ or MRP14^low^ (**d**). Original magnifications ×10, *scale bar* represents 200 μm, insets ×100

As IFA does not contain mycobacteria that were previously shown to be involved in IL-1β production [60], we also studied the expression of IL1β in brain tissue of animals immunized with rhMOG in CFA. We studied 432 perivascular lesions and 10 large areas with strong MHC class II expression and demyelination. IL-1β staining was observed in 36 % of the perivascular infiltrates (Fig. 2a) and in 70 % of the large areas with strong MHC class II expression and demyelination (Fig. 2b). Although IL-1β^+^ cells and MRP14^high^ cells were observed in close vicinity in the same lesions, all IL-1β^+^ cells were Iba-1^+^ (Fig. 2c) and MRP14^−^ or MRP14^low^ (Fig. 2d), similar to what was observed in animals immunized with rhMOG in IFA. In conclusion, IL-1β expression was associated mainly with MHC class II expressing cells present in perivascular infiltrates or at the edges of actively demyelinating lesions and not with infiltrating monocytes. Despite the fact that animals immunized with rhMOG in CFA were characterized by a much more rapid onset of clinical disease than those immunized with rhMOG in IFA, the IL-1β staining patterns were similar.

### MS

We started our characterization of IL-1β in MS by examining well-characterized paraffin-embedded tissue blocks of five donors without neurological disease and of 17 MS patients. MS lesions were characterized for the presence of demyelination by staining for PLP and for inflammation by staining for MHC class II and categorized as active, chronic active, and inactive [55, 61, 62]. Most tissue blocks contained multiple lesions of different categories (Table 5).

**Table 5:** IL-1β expression in paraffin-embedded sections of MS patients

	Total # lesions	Microglia nodules		Active lesions	Chronic active lesions	Inactive lesions
MS patients		Total	# IL-1β^+^			
1	10	5	–	2	1	2
2	1	0	–	1	0	0
3	6	3	–	1	2	0
4	4	3	–	1	0	0
5	19	10	4	4	5	0
6	1	0	–	1	0	0
7	14	0	–	6	8	0
8	3	0	–	2	1	0
9	11	0	–	11	0	0
10	2	0	–	1	1	0
11	6	3	3	1	2	0
12	9	1	–	1	3	4
13	7	6	–	0	0	1
14	8	5	–	3	0	0
15	4	0	–	1	2	1
16	2	2	1	0	0	0
17	6	0	–	3	3	0
Total	113	38	8	39	28	8

**Fig. 3:**
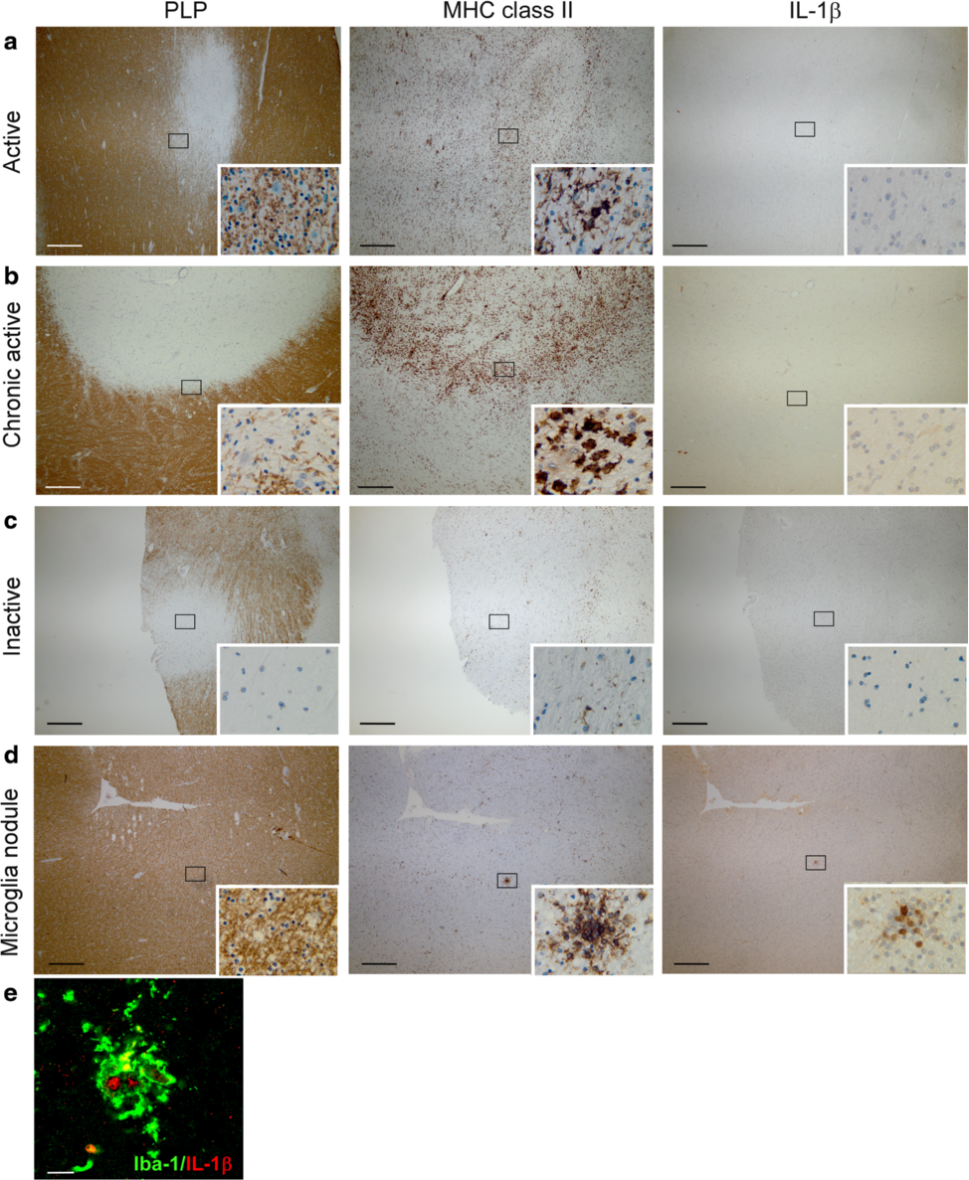
IL-1β expression in different types of MS lesions. MS lesions in paraffin-embedded brain tissue sections were characterized based on the extent of myelin (PLP *in brown, left panels*) damage and activation of innate immune cells (MHC class II *in brown, middle panels*). Active MS lesions were classified as areas with ongoing demyelination and activation of MHC class II^+^ innate immune cells (**a**). Chronic active lesions were classified by the presence of a completely demyelinated (PLP^−^) center surrounded by a rim of MHC class II^+^ cells (**b**). Inactive lesions were classified by the presence of demyelinated areas where the immune response has resided (**c**). We did not detect IL-1β (*in brown, right panels*) in active, chronic active, and inactive MS lesions in paraffin-embedded tissue sections (**a**–**c**). In addition, we observed MHC class II^+^ microglia nodules in otherwise NAWM (**d**) in which IL-1β was expressed. Double labeling of these microglia nodules for IL-1β (*in red*) and Iba-1 (*in green*) implicated that all IL-1β^+^ cells were Iba-1^+^ (**e**). Original magnifications ×4, insets ×100 (**a**–**d**), *scale bar* represents 500 μm (**a**–**d**) or 10 μm (**e**). Nuclei were counterstained with hematoxylin (*blue*; **a**–**d**)

We did not observe IL-1β staining in healthy controls. In contrast to our expectation, we also did not detect IL-1β expression in active, chronic active, or in inactive MS lesions (Table 5, Fig. 3a–c). Surprisingly, examination of normal appearing white matter (NAWM) from MS patients revealed IL-1β expression in nodules of MHC class II^+^ microglia (Fig. 3d). These microglia nodules occurred without evident signs of demyelination or infiltration and were previously described by different research groups [55, 61–63]. Although their role in MS pathogenesis is unclear at present, some authors suggested that these nodules are preactive lesions [55, 61, 62]. In total, we studied 38 of such microglia nodules in nine patients. IL-1β staining was observed in eight of the 38 microglia nodules (21 %; Table 5, Fig. 3d). The number of IL-1β^+^ microglia nodules varied between patients. In one patient, we observed exclusively IL-1β^+^ microglia nodules. In two patients, we observed both IL-1β^+^ and IL-1β^−^ microglia nodules, and in six patients, we observed exclusively IL-1β^−^ microglia nodules. Formal confirmation of the identity of the IL-1β^+^ cells as microglia was obtained by colocalization with Iba-1 (Fig. 3e). Irrespective of the immunization protocol, no equivalent of these microglia nodules was found in rhesus macaques with clinical EAE.

The existing literature on IL-1β expression in MS lesions contains contradicting observations. Whereas some studies reported a paucity of staining as we do [50, 51], others reported more extensive staining [47–49]. Differences in fixation procedures, tissue treatment and staining protocols may all have influenced the results. We therefore also characterized IL-1β staining in snap-frozen tissue blocks. For validation purposes, we included patients of which paraffin-embedded tissue sections had already been characterized by us.

**Fig. 4:**
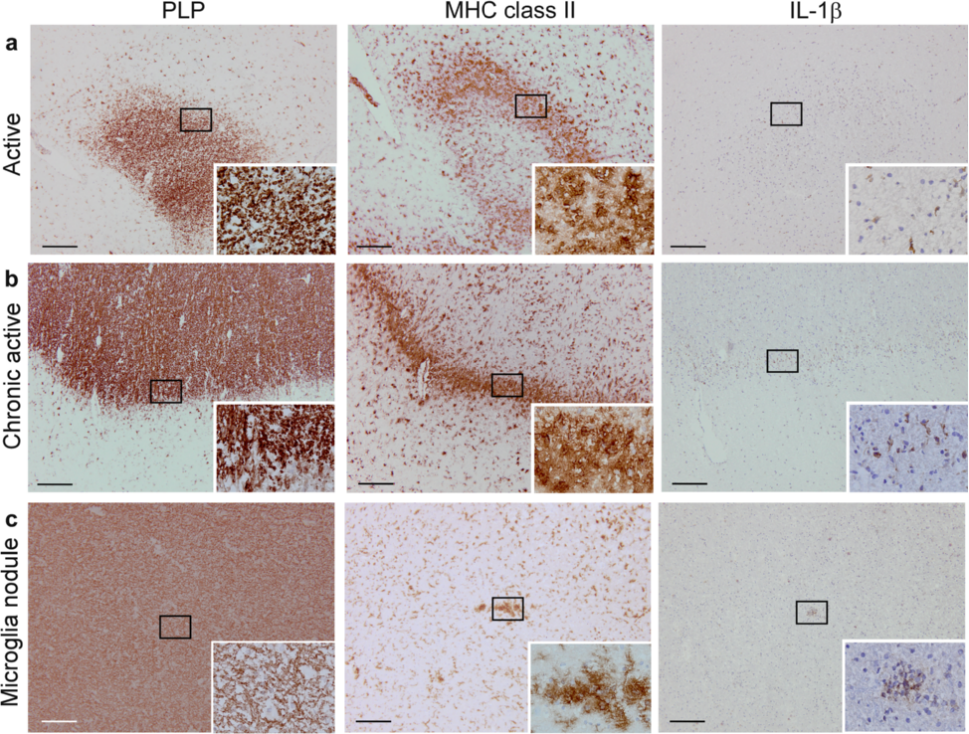
IL-1β expression in different types of MS lesions. MS lesions in frozen brain tissue sections were characterized based on the extent of myelin (PLP *in brown, left panels*) damage and activation of innate immune cells (MHC class II *in brown, middle panels*). Active MS lesions were classified as areas with ongoing demyelination and activation of MHC class II^+^ innate immune cells (**a**). IL-1β expression (*in brown, right panels*) was mainly observed in ramified MHC class II^+^ cells in the parenchyma, which were mainly localized at the edges of active lesions. Chronic active lesions were classified by the presence of a completely demyelinated (PLP^−^) center surrounded by a rim of MHC class II^+^ cells (**b**). IL-1β expression was observed in MHC class II^+^ cells in the rim of the lesion. Again, we observed MHC class II^+^ microglia nodules in otherwise NAWM (**c**) in which IL-1β was expressed. Original magnifications ×10, insets ×40 (**a–c**), *scale bar* represents 200 μm

In total, we studied 25 active lesions in 15 patients and six chronic active lesions in two patients. IL-1β staining in cryosections was more extensive than in paraffin-embedded sections, now also revealing expression in active and chronic active lesions. IL-1β staining was observed in 52 % of active lesions (Table 6, Fig. 4a). In nine patients, we observed IL-1β staining in ramified MHC class II^+^ cells in the parenchyma, whereas in six patients, we could not detect IL-1β in any of the active lesions. Similarly, IL-1β staining was observed in ramified MHC class II^+^ cells in the rim of all chronic active lesions (Table 6, Fig. 4b). In line with our earlier results, we also observed IL-1β staining in MHC class II^+^ microglia nodules in otherwise NAWM. In total, we studied 106 microglia nodules in seven patients. IL-1β staining was observed in 52 of these microglia nodules (49 %; Table 6, Fig. 4c). The number of IL-1β^+^ microglia nodules varied between patients. In one patient, all microglia nodules were IL-1β^+^, in three patients, we observed both IL-1β^+^ and IL-1β^−^ microglia nodules, and in three patients, we observed only IL-1β^−^ microglia nodules.

**Table 6:** IL-1β expression in frozen sections of MS patients

	Total # lesions	Microglia nodules		Active lesions		Chronic active lesions		Inactive lesions	
MS patient		Total	# IL-1β^+^	Total	# IL-1β^+^	Total	# IL-1β^+^	Total	# IL-1β^+^
3	2	0	–	2	2	0	–	0	–
5	7	2	2	3	3	2	2	0	–
8	6	4	1	1	1	0	–	1	–
11	3	2	–	1	1	0	–	0	–
16	80	75	41	1	1	4	4	0	–
18	22	20	8	2	2	0	–	0	–
19	3	1	–	2	1	0	–	0	–
20	1	0	–	0	–	0	–	1	–
21	1	0	–	1	–	0	–	0	–
22	1	0	–	1	1	0	–	0	–
23	6	0	–	6	1	0	–	0	–
24	3	2	–	1	–	0	–	0	–
25	1	0	–	1	–	0	–	0	–
26	1	0	–	1	–	0	–	0	–
27	1	0	–	1	–	0	–	0	–
28	2	0	–	1	–	0	–	1	–
Total	140	106	52	25	13	6	6	3	0

**Fig. 5:**
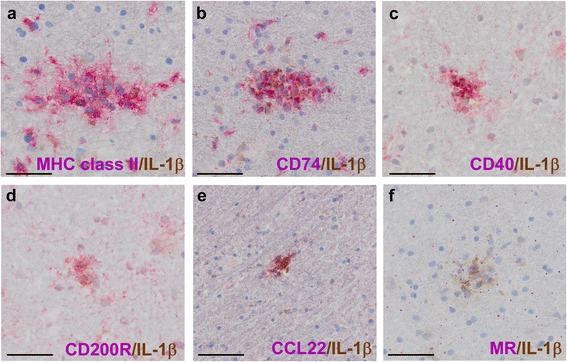
Activation status of IL-1β^+^ cells in microglia nodules. Microglia nodules were classified as clusters of MHC class II^+^ cells in otherwise NAWM. These clusters of activated microglia were double stained for IL-1β (*in brown*) and cell surface markers associated with pro-inflammatory or anti-inflammatory cellular phenotypes (*in red*). IL-1β staining colocalized with MHC class II (**a**) and cell surface markers CD74 (**b**) and CD40 (**c**) as well as with CD200R (**d**). In most microglia nodules, IL-1β staining also colocalized with CCL22 (**e**), although some microglia nodules contained IL-1β^+^/CCL22^−^ cells. IL-1β^+^ microglia did not express MR (**f**). Original magnifications: ×40, *scale bar* represents 50 μm. Nuclei were counterstained with hematoxylin (*blue*)

To further characterize the IL-1β^+^ cells, microglia nodules were stained for molecules associated with pro- and anti-inflammatory phenotypes [64]. IL-1β staining in MHC class II^+^ microglia nodules (Fig. 5a) colocalized with the pro-inflammatory markers CD74 (Fig. 5b) and CD40 (Fig. 5c) as well as with the anti-inflammatory marker CD200R (Fig. 5d). In most microglia nodules, IL-1β staining also colocalized with the anti-inflammatory marker CCL22, although some microglia nodules contained IL-1β^+^/CCL22^−^ cells (Fig. 5e). By contrast, IL-1β^+^ microglia did not stain for mannose receptor (MR; Fig. 5f). As previously described [64, 65], MR staining was predominantly observed in perivascular spaces and not in microglia nodules. IL-1β^+^ and IL-1β^−^ microglia nodules could not be distinguished based on the expression of these markers. In conclusion, IL-1β^+^ nodular microglia expressed a mix of pro-inflammatory and anti-inflammatory markers, in line with other reports [64].

Microglia in the rim of chronic active lesions expressed a similar mix of pro- and anti-inflammatory markers as the microglia in the nodules (Additional file 1), except that not all IL-1β^+^ cells in the rim of chronic active lesions were CD74^+^ and that more colocalization was found with CCL22, which may be indicative of a slightly less pro-inflammatory profile.

**Fig. 6:**
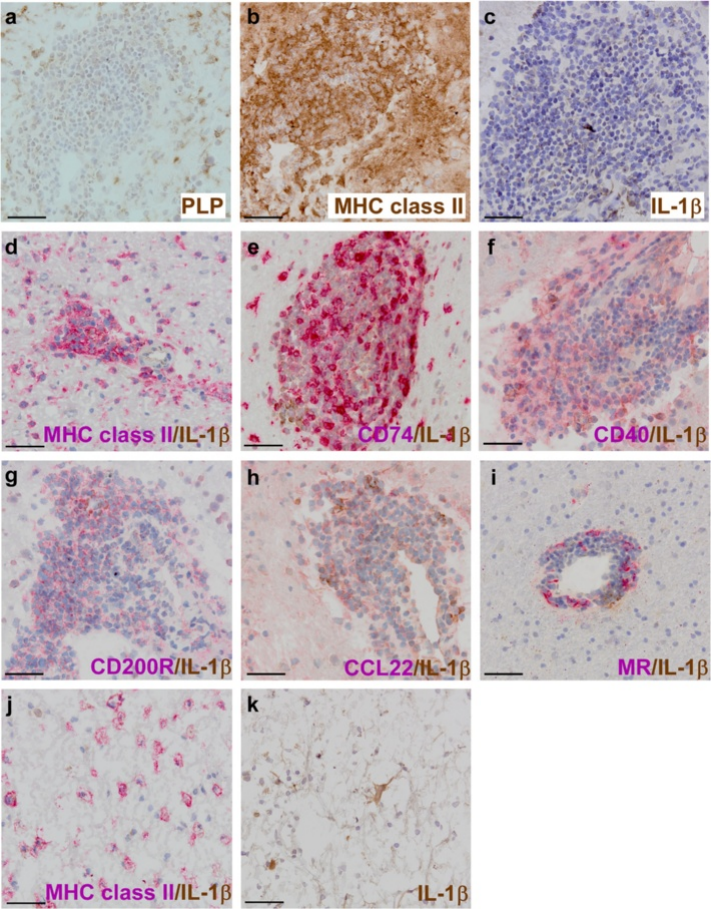
IL-1β expression in perivascular infiltrates in active MS lesions. Within the active lesions of two patients, we observed perivascular infiltrates in areas with ongoing demyelination (PLP, *in brown*; **a**). These perivascular cells were strongly MHC class II^+^ (*in brown*; **b**). We observed IL-1β expression (*in brown*) in cells associated with these perivascular infiltrates, mainly at the edges of the infiltrate and the parenchyma (**c**). These cells were double stained for IL-1β (*in brown*) and cell surface markers associated with pro-inflammatory or anti-inflammatory cellular phenotypes (*in red*). IL-1β staining colocalized with MHC class II (**d**) and CD74, although we also observed multiple IL-1β^+^/CD74^−^ cells (**e**). Furthermore, IL-1β staining colocalized with CD40 (**f**), CD200R (**g**), and CCL22, although we also observed some IL-1β^+^/CCL22^−^ cells (**h**). IL-1β and MR staining were both observed in the same perivascular infiltrates, but all IL-1β^+^ cells were MR^−^ (**i**). In addition, within one active lesion, we observed MHC class II^+^ cells (*in red*) with a foamy appearance (**j**). These MHC class II^+^ foamy cells did not express detectable levels of IL-1β. In addition, we observed some IL-1β staining (*in brown*) in reactive astrocytes in the same active lesion (**k**). Original magnifications ×40, *scale bar* represents 50 μm. Nuclei were counterstained with hematoxylin (*blue*)

Since IL-1β expression in EAE was mainly localized to perivascular infiltrates, we screened our available patient material for such lesions. In two patients, very active lesions were found that were associated with large perivascular infiltrates. Here, we also observed IL-1β staining in cells associated with the perivascular infiltrates (Fig. 6a–c), mainly at the edges of the infiltrates. These cells were MHC class II^+^ (Fig. 6d) and again expressed a mix of pro- and anti-inflammatory markers (Fig. 6e-h). Although IL-1β and MR staining were observed in the same perivascular infiltrates, IL-1β staining never colocalized with MR staining (Fig. 6i), nor with MHC class II^+^ cells with a foamy appearance (Fig. 6j). Although this may suggest that myelin ingestion inhibits the production of IL-1β, as was shown previously for other pro-inflammatory cytokines [50], we could not confirm this in vitro (data not shown). We did also observe some IL-1β immunoreactivity in reactive astrocytes (Fig. 6k), as reported by other authors [47]. In both patients that showed these IL-1β^+^ perivascular lesions, microglia nodules and ramified cells within the rim of chronic active lesions were also IL-1β^+^.

## Discussion

Different lines of evidence suggest that IL-1β has a pathogenic role in MS and in the animal model for MS, EAE [26, 28, 32, 35]. Here, we characterized the expression of IL-1β in brain tissue from rhesus macaques with EAE and in different MS lesion types. Contrary to our expectations, we observed that IL-1β expression was mainly restricted to glia cells, most importantly microglia, both in EAE as well as in MS. In rhesus EAE, IL-1β expression was most abundant in perivascular lesions and in active demyelinating lesions with large infiltrates, whereas in MS IL-1β expression was much less abundant and mainly observed in parenchymal nodules of activated microglia.

Although the perivascular localization of IL-1β in rhesus EAE was largely in line with previous studies in rodents [29, 30, 46] and in accordance with the peripheral induction of disease, we did not detect IL-1β in MRP14^high^ monocytes that had recently infiltrated the CNS or in T lymphocytes [31, 66]. Especially in animals immunized with rhMOG in CFA, the enhanced immunogenicity caused by the presence of mycobacteria in the adjuvant has been linked to their ability to directly cause IL-1β expression, inflammasome activation, and IL-1β secretion in monocytes and macrophages [60, 67–69]. The induced expression of pro-IL-1β by immunization is however local and most likely of a transient nature. Recently, we described that in vitro pro-IL-1β expression can be potently induced in rhesus macaque primary microglia and peripheral macrophages, but that expression is subject to strong and rapid negative regulation [70]. As the last immunization was performed at least 12 days before euthanasia, it is unlikely that the immunization-induced expression of IL-1β is responsible for the staining pattern observed in the CNS. Our results suggest that IL-1β expression is induced within the CNS and reflects a tissue response to stress that is associated with infiltration of peripheral immune cells. This would also be in line with the IL-1β^+^ microglia in brain tissue of animals immunized with rhMOG in IFA. We are not the first to report on this phenomenon, as previous studies demonstrated that IL-1β expression in microglia-like cells is increased by infiltration of immune cells into the CNS [71] and that NLRP3 inflammasome activation is induced in rodents where EAE was passively induced [35]. Although different studies have reported on the expression of IL-1β in infiltrating T lymphocytes in rodent EAE [31, 66], we did not detect IL-1β^+^ T cells in rhesus EAE tissue. This discrepancy may be attributable to differences in immunization protocols or to differences between species. In this context, it is noteworthy that the rhesus EAE model is characterized by a hyperacute development of clinical symptoms, rendering the model less suitable to study more chronic features of the neuroinflammatory process. The marmoset EAE model might provide a suitable alternative for further studies on this topic as it is characterized by a more chronic development of clinical symptoms [72].

IL-1β expression in MS was much less prominent as in rhesus EAE and the staining pattern was markedly different. In MS, IL-1β expression was mainly localized in the parenchyma, especially in parenchymal nodules of activated microglia. We observed that only a portion of these nodules was IL-1β^+^. Characterization of the IL-1β^+^ microglia in these nodules using markers for anti- and pro-inflammatory phenotypes showed that these cells express a mix of both markers, which is in line with other studies [64, 65]. It has been proposed that most of these nodules might resolve spontaneously while other might progress into an active lesion [61], and previous studies have demonstrated that IL-1β can initiate the demyelination process [73, 74]. Whether the expression of IL-1β is a discriminating factor regarding the fate of the nodules remains to be determined as it is also well possible that the microglial expression of IL-1β merely reflects a transient response to cellular stress or to neuronal degeneration [75]. Various molecules associated with acute cellular stress induce IL-1β expression, including IL-1α, TNFα, the small stress protein alphaB-crystallin (HspB5), and high mobility group box 1 (HMGB1) [9, 76, 77]. Interestingly, HspB5 and TNFα are expressed in microglia nodules in MS [55, 76] and may contribute to the IL-1β expression as described here. However, whether these factors are specifically associated with the IL-1β^+^ microglia nodules remains to be investigated.

Interestingly, IL-1β has recently been demonstrated to play a role in neuronal degeneration via a p53-mediated apoptotic cascade [78]. In addition, IL-1β might affect cortical excitability in MS patients [43] and can be detected in the gray matter of rats in which chronic-relapsing EAE was induced [29]. We have therefore also analyzed IL-1β expression in five leukocortical lesions that were present in four patients. In the limited number of lesions we studied, IL-1β expression was barely detectable and almost exclusively restricted to the white matter (data not shown). A possible explanation might be that most cortical demyelination is thought to occur early during MS pathogenesis [79], and inflammatory activity might already have resolved in the lesions we studied. This topic warrants further investigations, both in MS and in EAE. Again, the rhesus EAE model is not suitable for such a study, as gray matter lesions are not present.

The etiology of MS is still debated, and both infectious and non-infectious factors have been proposed as inducers or precipitators of the disease [80–82]. NLR activation has been reported in response to infectious and sterile inflammation, and inflammasome-induced IL-1β might represent an a-specific hallmark of disrupted brain homeostasis, both in EAE and in MS. However, in contrast to MS, we did not observe IL-1β^+^ microglia nodules in rhesus EAE, which is most probably due to the acute nature of the model. Whether IL-1β expression as observed in MS can also be observed in more chronic EAE models requires further study.

## Conclusions

In conclusion, the expression pattern of IL-1β in EAE and MS is consistent with a response that is initiated in the tissue rather than with the infiltration of IL-1β-producing monocytes. Whether this response plays a role in the exacerbation of the disease remains to be demonstrated. Most importantly, we here describe that a subpopulation of parenchymal IL-1β^+^ microglial nodules can be distinguished exclusively in MS with an as yet unknown role in lesion initiation or progression.

## Additional file

Additional file 1: Figure S1 Activation status of IL-1β^+^ cells in the rim of chronic active lesions. IL-1β^+^ cells in the rim of chronic active lesions were double stained for IL-1β (*in brown*) and cell surface markers associated with pro-inflammatory or anti-inflammatory cellular phenotypes (*in red*). IL-1β staining colocalized with MHC class II (**a**) and with CD74 (**b**), although we also observed some IL-1β^+^/CD74^−^ cells. Furthermore, IL-1β staining colocalized with CD40 (**c**), CD200R (d), and CCL22 (**e**), although we also observed some IL-1β^+^/CCL22^−^ cells. IL-1β and MR staining were both observed in rim of chronic active lesions (**f**), but all IL-1β^+^ cells were MR^−^. Original magnifications ×40, *scale bar* represents 50 μm. Nuclei were counterstained with hematoxylin (*blue*). (TIF 7190 kb)
