# Supplementary figures and images for: Expression of IL-1β in rhesus EAE and MS lesions is mainly induced in the CNS itself
Source: J Neuroinflammation. 2016 Jun 6;13:138. doi: 10.1186/s12974-016-0605-8 (PMC4895983; doi:10.1186/s12974-016-0605-8)

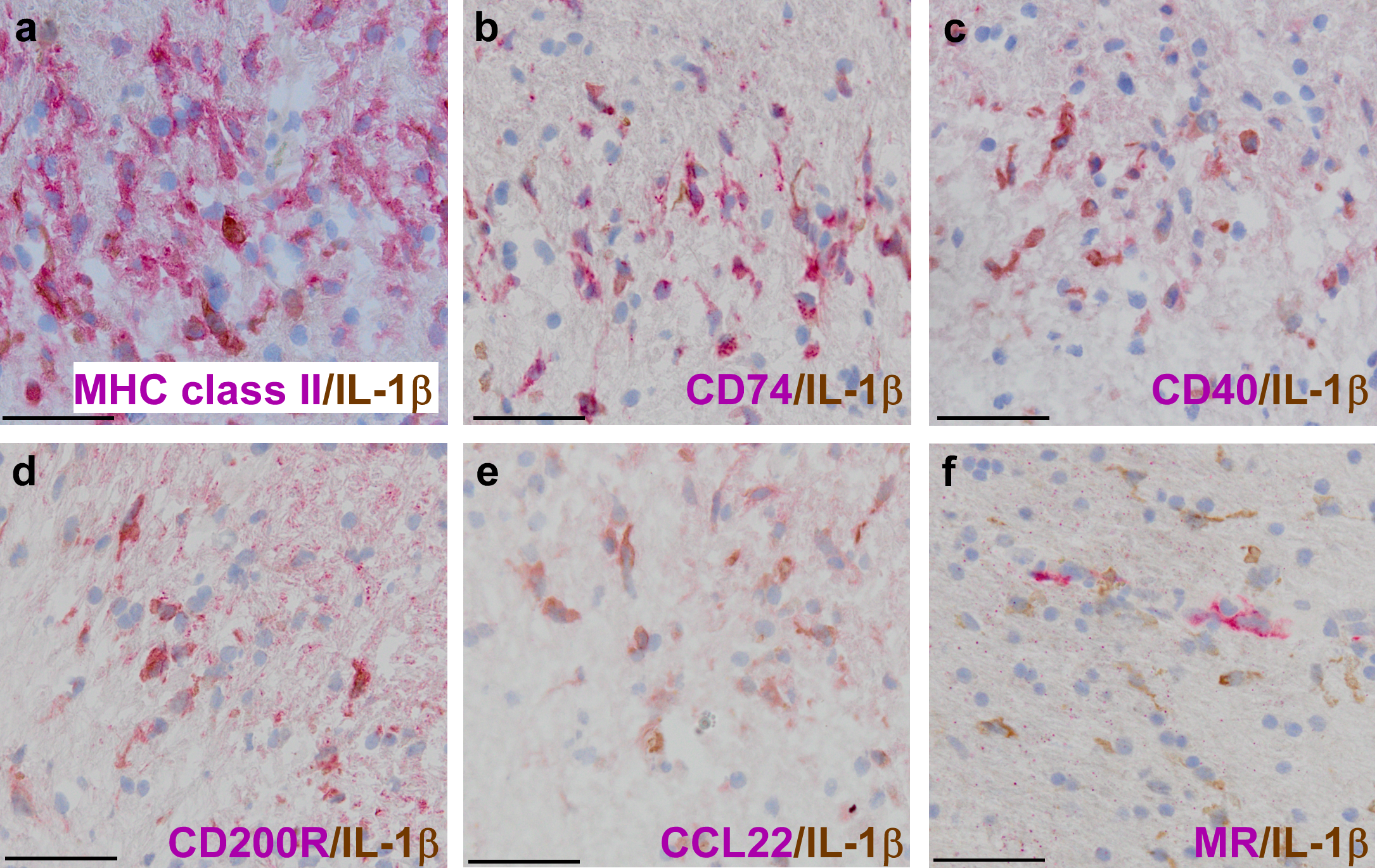

Supplement: Additional file 1: Figure S1 — Activation status of IL-1β+ cells in the rim of chronic active lesions. IL-1β+ cells in the rim of chronic active lesions were double stained for IL-1β (in brown) and cell surface markers associated with pro-inflammatory or anti-inflammatory cellular phenotypes (in red). IL-1β staining colocalized with MHC class II (a) and with CD74 (b), although we also observed some IL-1β+/CD74− cells. Furthermore, IL-1β staining colocalized with CD40 (c), CD200R (d), and CCL22 (e), although we also observed some IL-1β+/CCL22− cells. IL-1β and MR staining were both observed in rim of chronic active lesions (f), but all IL-1β+ cells were MR−. Original magnifications ×40, scale bar represents 50 μm. Nuclei were counterstained with hematoxylin (blue). (TIF 7190 kb) [file 12974_2016_605_MOESM1_ESM.tif]
